# Comparison of Two Surgical Methods, Primary Closure and Rotational Flap, in Patients With Chronic Pilonidal Sinus

**DOI:** 10.5539/gjhs.v6n7p18

**Published:** 2014-09-18

**Authors:** A. Enshaei, S. Motearefi

**Affiliations:** 1 Urmia University of Medical Sciences and Health Services, Urmia, Iran

**Keywords:** asymptomatic sinus, dehiscence, hematoma, pilonidal sinus, rotational flap

## Abstract

**Introduction:**

Pilonidal sinus is a common infectious process which occurs in buttocks and sacral area which involves a wide range of symptoms that are different from an asymptomatic sinus abscess to acute and chronic sinus track. Basically non-surgical treatment for this disease is not recommended. In this study, we have been compared two methods of primary repair and rotation flap in terms of factors such as duration of hospitalization, recurrence, infection etc.

**Methods:**

80 patients with chronic pilonidal sinus were randomly divided into two groups and underwent surgery. Diabetic and obese patients with underlying diseases and patients with acute pilonidal abscess or prior surgery were excluded. The patients’ hospital stay, duration of postoperative pain, itching and hematoma, were investigated.

**Results:**

In this study, patients’ sex and mean age were examined in terms of frequency of complications of hematoma, wound infection, recurrence, itching, and duration of hospitalization and the presence of seroma, there is no significant difference between the two methods of primary surgical repair and rotation flap (P>0.05) But in terms of the opening of the surgical wound, in primary surgical method, 5 patients (12.5%), wound dehiscence were reported, in rotation flap, any case of wound dehiscence were reported. There is significant difference between wound dehiscence in patients with chronic pilonidal sinus and two methods of surgery. (P=0.02). The mean duration of pain relief was 15.2±3.35 days in the primary surgical repair method and rotation flap was 7±2.3 days. According to the test there is significant difference between mean duration of pain relief and two surgical methods. (P=0.001). The mean duration of sutures was 15.3±2.3 days in the primary repair method and in rotational flap was 12±3.6 days. There is significant difference between the mean duration of sutures and two surgical methods (P=0.001)

**Conclusion:**

Considering these results, rotational flap is the preferred method due to fewer complications, lower recurrence after surgery and faster healing time of surgical wounds and as a result, the effective force’s early return to economic cycle. Finally, we can say that each surgeon according to the type and size of the sinuses and occupational status and social class, personality and individuality of the patient can select the appropriate method of surgery.

## 1. Introduction

Pilonidal sinus is a common infectious process which occurs in buttocks and sacral area which involves a wide range of symptoms that are different from an asymptomatic sinus abscess to acute and chronic sinus track (De Parades, Bouchard, Janier, & Berger, 2013). The disease was first described in 1833 by Mao. The disease occurs mostly in young adults and its incidence is 26 cases per 100,000 and in men is twice women, the peak incidence was between 15 and 24 years of age and rarely occurs after age 40 (McCallum, King, & Bruce, 2004; Raffas & Hassam, 2013). The etiology of this disease is not fully understood, some are believed to be congenital in origin, and some consider it an acquired disease and the reason to this is that this condition can be seen in folds between the fingers of hairdressers and shepherds and dog trainers which can be due to the penetration of the hair as a foreign body and cause reactions in the subcutaneous tissue (Bradley, 2010a, 2010b). Hull et al. have suggested that hair in this area come in clusters and like a drill bit into the skin and gradually in-depth down (Hull & Wu, 2002). Sondenna and et al. have proposed that during puberty, due to rapid growth gluteus muscles, distance of hair follicles, sebaceous glands and apocrine glands increases and this event increases the probability of foreign body entrance into the skin and causes holes, which is the precursor of the formation of pilonidal sinus (Sondenaa et al., 1996). The sinuses are asymptomatic until they are not infectious and when they become infected the symptoms of intermittent pain, tenderness, and intermittent serous-purulent fluids manifest. Other manifestations include sinus abscess that is acute and is at the upper and outer left position than vents (Chintapatla, Safarani, Kumar, & Haboubi, 2003). Primarily non-surgical treatment for this disease is not recommended and is only recommended for patients who have the least symptoms such as hygiene sacrococcygeal region and shaving regional hair weekly. There are several methods for surgical treatment that the method should be appropriate for each individual with clinical features of the disease. The ideal treatment should be the minimum required hospitalization after surgery, be simple, with minimal pain, the patient soon returned to his work, minimal risk of complications and should be treated easily in case of the recurrence. Various therapeutic approaches, each with advantages and disadvantages are presented in recent years (Rabie, 2007; Sondenaa et al., 2002). In acute abscesses the treatment is as incision and drainage and elective mode of treatment for chronic pilonidal sinus as incision and primary repair, incision and flap (rotational - rhomboid and Z plasticizer), incision and leaving the wound open to repair secondary, due to prolonged wound healing and return to work in the classical surgery (open surgery) and according to the few studies in terms of comparing the two methods of primary repair and rotational flap, it is necessary to compare the two methods, so we aimed to conduct a comparative study between surgical primary repair and rotational flap in terms of factors, such as duration of hospitalization, the recurrence rate, rate of infection, itching or hematoma, the time period of sutures and wound dehiscence.

## 2. Methods

The study design has been done as experimental clinical trial after research and ethics committee approval, patients with pilonidal sinus who had not previously undergone surgery for this disease were enrolled without limitations on age and sex. Chronic pilonidal sinus is defined as the presence of secretion and pain in sacrococcygeal region that is not formed abscess. The number of sample was 80. Patients were randomly divided into two groups of 40. Diabetic and obese patients with underlying diseases and patients with acute pilonidal abscess or prior surgery were excluded. All patients before surgery their hair was removed by depilatory. In primary repair group, the skin after removal of affected area in sacrococcygeal area up to the posterior sacral fascia after making adequate hemostasis has attempted to repair with nylon 0.2, so that the edges are closer together. In rotational flap after resection of the affected area formed the subcutaneous skin flap with a proper pedicle flap in the proximal area and by turning the flap into defect area come closer with minimal tension. Antibiotics were administered to patients before and after surgery, but analgesics were administered as needed. Then, depending on the clinical status, patients discharged and the first and fourth weeks and 6 months postoperatively were visited and questionnaires were completed. For the analysis of the present study SPSS 16 software is used (Rezaie et al., 2013).

## 3. Results

In terms of sex 22 patients (55%) were male and 18 patients (45%) were female in primary surgical repair and 29 patients (72.5%) were male and 11 patients (27.5%) were female underwent surgical flap. According to the chi-square test, there is no significant difference between sexes in two surgical procedures (P=0.1). The mean age of the patients was 24.17± 6.05 minimum 15 and maximum 40.

Frequency of hematoma in pilonidal sinus in primary repair method in 1 patient (2.5%) and in rotational flap in 3 patients (7.5%) minor hematoma was reported and in 37 patients (92.5%) cases of hematoma were not reported. According to Fisher Exact test there is no significant difference in terms of the frequency of hematoma in two surgical procedures (P=0.3) (Table 1).

**Table 1:** Distribution of absolute and relative frequency of complications and hospitalization of pilonidal sinus surgery using both primary repair and rotational flap methods

Variable	Primary repair method	Rotational flap	P. value
Hematoma			
Have	(%2.5)1	(%7.5)3	0.3
Not have	(%97.5)39	(%92.5)37	

Infection			
Have	(%2.5)1	(%0)0	0.3
Not have	(%97.5)39	(%100)40	

Recurrence			
Have	(%2.5)	(%0)0	0.3
Not have	(%97.5)39	(%100)40	

Itching			
Have	(%10)4	(%0)0	0.05
Not have	(%90)36	(%100)40	

Seroma			
Have	(%2.5)1	(%7.5)3	0.3
Not have	(%97.5)39	(%92.5)37	
Dehiscence			
Have	(%12.5)5	(%0)0	0.02
Not have	(%87.5)35	(%100)40	

Hospitalization			
One day	(%97.5)39	(%92.5)37	0.3
Two days	(%2.5)1	(%7.5)3	

Of 40 patients with primary repair method, 39 patients (97.5%) for one day, and 1 patient (2.5%) for 2 days were hospitalized and in rotational flap, 37 patients (92.5%) for one day and 3 patients (7.5%) for two days were hospitalized. According to Fisher Exact test there is no significant difference between the duration of hospitalization in two surgical procedures (P=0.3) (Table 1).

Of 40 patients with primary repair method, 1 patient (2.5%) had wound infection and in 39 patients (97.5%), wound infection was not reported. In rotational flap, wound infection was not reported. According to Fisher Exact test there is no significant difference between the incidence of infection in patients with chronic pilonidal sinus and two surgical procedures (P=0.31) (Table 1).

Of 40 patients with primary repair method, 1 patient (2.5%) had recurrence and in 39 patients (97.5%) recurrence was not reported. In rotational flap the cases of recurrence were not reported. According to Chi-square test there is no significant difference between the risks of recurrence in patients with chronic pilonidal sinus and two surgical procedures. (P=0.31) (Table 1).

Of 40 patients with primary repair method, 4 patients (10%) had itching, and 36 patients (90%) did not itch. And in rotational flap the cases of itching were not reported. According to the Fisher Exact test there is no significant difference between patients’ complain of itching in patients with chronic pilonidal sinus and two surgical procedures. (P=0.05) (Table 1).

Of 40 patients with primary repair method, in 1 patient (2.5%) seroma was reported and 39 patients (97.5%) had not seroma. In rotational flap, in 3 patients (7.5%) seroma was reported and in 37 patients (92.5%), seroma were not reported. According to Fisher Exact test there is no significant difference between the presence of seroma in patients with chronic pilonidal sinus and two surgical procedures. (P=0.3) (Table 1).

Of 40 patients with primary repair method, in 5 patients (12.5%), wound dehiscence were reported and in 35 patients (87.5%) cases of wound dehiscence were not reported. In rotational flap the cases of wound dehiscence were not reported. According to the Fisher Exact test, there is a significant difference between dehiscence in patients with chronic pilonidal sinus and two surgical procedures. (P=0.02) (Table 1).

The mean duration of pain relief in patients treated with primary repair method was 15.2±3.35 days and in patients with rotational flap was 7±2.3 days. According to the t-test there is a significant difference between the mean duration of pain relief in patients and two surgical procedures. (P=0.001) (Table 2).

**Table 2:** Comparison of mean and standard deviation for pain relief, time sutures in the study population

Surgery method Variable	Primary repair	Rotational flap	P.value
Mean and standard deviation of pain relief (day)	3.35 ± 15.2	2.3 ± 7	0.001
Mean and standard deviation of suture (day)	2.3 ± 15.3	3.6 ± 12	0.001

The mean duration of sutures in patients treated with primary repair method was 15.3±2.3 days, and in patients treated with rotational flap was 12±3.6 days. According to the t-test there is a significant difference between the mean duration of sutures in patients and two surgical procedures (P=0.001) (Table 2).

## 4. Discussion

Pilonidal sinus in men is twice women (McCallum et al., 2004; Raffas & Hassam, 2013). In our study the incidence of pilonidal sinus in males is more than females (63.8%) and mostly young adult patients with a mean age of 24.17±6.05 years. According to the results of the study, in terms of the complications after surgery, such as frequency of hematoma and seroma, wound infection, itching, recurrence and hospitalization time there was no significant difference between the two rotational flap method and primary repair. The results of Okus et al. study (Okus, 2012) in 2012 on 49 patients with flap and 44 patients underwent primary repair surgery in terms of mean age (25 years) and no recurrence during 6-month follow-up in both group and one case of wound infection in the primary repair group was consistent. And also with results of Nessar et al. (2004) study in teaching hospital in Ankara in 2014 on 20 patients who underwent rotational flap, mean age of patients was 23.4 years, all patients were discharged the day after surgery and within two weeks had complete healing and no wound infection and no recurrence was observed was consistent. In a study by Daphan et al. (2004) in 2004 on 147 patients who had undergone surgical flap, recurrence was reported in 7 cases that were not consistent with our results, but no cases of infection have been reported, three patients had seroma, which was consistent with our results.

But in the mean duration of pain relief in flap rotation (7±2.3) with primary repair (15.2±3.35) and the mean duration of sutures in patients with rotation flap (12±3.6) with primary repair patients (15.3±2.3) and the frequency of surgical wound dehiscence at the flap rotation (0%) with primary repair (5 cases), a significant difference was perceptible. The results could be due to the high elasticity of skin and subcutaneous tissue caused by closure of these tissues by suture in primary repair. Although the elasticity of the tissues is much lower in rotational flap method. The results in these cases are consistent with the results of Nessar (2004), but with the result of Aslam et al. (2009) that was conducted in 2009 on 110 patients with pilonidal sinus that underwent flap, 2 patients with opening in flap and 1 patient partial necrosis flop and 3 cases infection and the mean hospitalization was 3 days and one case of recurrence, was different.

## 5. Conclusions

Considering these results, rotational flap is the preferred method due to fewer complications, lower recurrence after surgery and faster healing time of surgical wounds and as a result, the effective force’s early return to economic cycle. The only point at issue in the case of rotational flap is dissatisfaction from scarring after surgery and anesthesia of the surgical site that in our study due to the small size of rotational flap these were not observed. Finally, we can say that each surgeon according to the type and size of the sinuses and occupational status and social class, personality and individuality of the patient can select the appropriate method of surgery.
